# Academy of Stomatology

**Published:** 1904-02

**Authors:** 

**Affiliations:** Academy of Stomatology


### ACADEMY OF STOMATOLOGY.

A regular meeting of the Academy of Stomatology, of Philadelphia, was held at the rooms of the Academy, 1731 Chestnut Street, on the evening of Tuesday, October 27, 1903, the President, Dr. L. Foster Jack, in the chair. A paper was read by Dr. P. B. McCullough, entitled "A Suspension Partial Denture;" also one by Dr. H. B. Hickman, entitled "Osteomyelitis: A Case in Practice."

(For Dr. McCullough's paper, see page 105; for Dr. Hickman's paper, see page 111.)

DISCUSSION.

*Dr. J. H. Gaskell.*—I have been very much interested in Dr. McCullough's paper, but I cannot but feel that it is a mistake to saddle or have a broad surface of the mucous membrane covered by a permanent fixture in the mouth. No matter how accurately it may be attached, the gum is a tissue which is more or less yielding, and the pressure is bound to force food between that fixture and the mucous membrane. If it is placed permanently the pressure will cause absorption, so that in time the gum will not be as firm as when the fixture is put into place. I have seen results from bridges made very similar to the one described by Dr. McCullough. I have had occasion to take one out which had been in for two years. The tissue had become infected, and was inflamed and swollen, and when I took off the bridge the periosteum was entirely exposed.

*Dr. William H. Trueman.*—The conditions we have to meet in crown- and bridge-work are so different that we need all the light we can obtain. There is no doubt that in many cases the method described by Dr. McCullough will answer a good purpose. It also has in my judgment some weak points. When put on a molar or bicuspid, my experience has always been that the apparatus gave way. In one case I had made a joint that I thought could not possibly open; nevertheless it did it very promptly. When the pressure is all exerted towards the gum such an attachment may answer for a long time, but it does not answer when side pressure is exerted. I always prefer to have some kind of a band around the tooth to hold it firmly. The point which the doctor makes about pressing the gum is a good one. A nice adaptation is made and the area of pressure is limited, and absorption does not take place to any great extent. I hardly think the attachment is safe made between the two bicuspid teeth, where we are told the strain exercised is several hundred pounds. It is always well to remember that in these cases it is the impossible that generally happens. Unless all the force of mastication is brought against the gold there is lack of durability, and in this respect the method shows a weak point. With the second bicuspid and molars it is wiser to dispense with the porcelain and make an open bridge. I am in the habit of doing this, and the method is satisfactory.

There are points in the doctor's paper that can only be taken in as we read and think over it. There are many cases in which the method has a practical application. We cannot tie ourselves to any one system, but must do the very best we know how for the patient.

*Dr. E. C. Kirk.*—I do not want to betray confidence, but would say that some years ago Dr. Henry C. Register showed me a bridge appliance which had one feature in common with those described by the essayist: the crowns fitted the mucous membrane very closely, and Dr. Register has evidently had experience as to the tolerance of this mucous tissue of the contact of foreign bodies. The method employed by the essayist seems to be, in a measure, a theoretical one after all. The tolerance of the gum tissue, which has been by one speaker condemned, has been by another approved.

*Dr. H. C. Register.*—I hesitate to talk upon the subject, but feel that I must express myself entirely in favor of small open bridge-work.

I commenced the bridging of teeth certainly eighteen or twenty years ago. I took off two saddle bridges this last winter that had been doing service for eighteen years. It is a decided mistake, in speaking of the saddle, not to define it very carefully. The saddle represented in Dr. McCullough's first figure is about the proper thing; it is absolutely clean. Many persons, in speaking of a saddle, mean a continuous saddle. I have used in certain cases a very narrow saddle, and have not confined it to the tooth, but made it a continuous one. Even in those cases I have never had any serious trouble.

I think Dr. McCullough's plan very desirable in its application to the bicuspids in these cases, as we find it very difficult without destroying two teeth to get sufficient anchorage. Teeth of this character placed in the mouth are much more comfortable to the patient than those made on the ordinary lines of bridge-work. In the case of the two bridges I spoke of I replaced with the more approved style of bridge-work, having the tip of the tooth touch the gum and without the lingual contour of the natural tooth. Both cases went to Europe, and I have received three or four letters from each saying how much more comfortable the old appliance was than the new.

*Dr. A. N. Gaylord.*—In my experience the so-called clearing space in bridge-work is in reality a place for the collection of food. Dr. McCullough's idea of fitting the tooth against the gum seems preferable. I have yet to see a bridge, where the surface bevelled from the inner cusp to the outer gum margin, which was not covered with more or less deposit. It is difficult, and in many cases impossible, to keep such a place clean, besides being annoying to the tongue. I have not received a sufficiently clear idea of the method of the essayist to endorse or speak against it. I would like to see a case that I might study it more fully. However, I am much in favor of removable bridges. I would ask Dr. McCullough the comparative time required in making this appliance and an ordinary bridge. I should imagine it much greater. If so, I should prefer to spend the extra time making a removable appliance, which could be cleansed in the hand and replaced. Such appliances are cleanly and satisfactory in every way.

*Dr. McCullough.*—Should a fair test of the principle here elucidated, of covering a limited area of gum tissue with a fixed denture, for pathological or mechanical reasons, prove impracticable, then the only change necessary to be made in the method of construction to insure success will be to make the surfaces of the teeth next the gum, mesio-distally, convex, and support the bridge anteriorily by a lug forming a movable joint with a filling in the cuspid.

This is the most conservative method, involving the cuspid, that can be used. By it the pulp is not involved, the natural independent mobility of the natural teeth is not impaired, and the bridge is more artistic, cleaner, and more acceptable to the patient than the modern bridge for the same space.

Presupposing the cuspid tooth sound, to substitute the two bicuspids without involving the cuspid leaves as the only alternative the resting of these teeth upon the gum.

When it is proved that the principle of covering the gum, as here interpreted, is unscientific, then I am unwilling to believe that any form of fixed saddle-bridge can have dental merit.

Justification for the belief in its possible practicability may be found in the following three cases of a number observed in practice.

One, showing the importance of imitating the forms of the natural teeth as a purely moral factor, is that of a woman for whom I made an upper vulcanite plate with five or six teeth. On the right side the only artificial tooth attached to the plate was the first molar. When this plate was placed in the mouth it seemed to have every point of merit that could be desired.

In a few days the patient returned and in a very positive manner stated that the plate did not fit.

After some fruitless questioning, I asked the patient, "On which side, in your opinion, is it that the plate does not fit? She indicated the right side. Here I observed that the buccal face of the artificial tooth extended out of line of the buccal faces of the two adjacent natural teeth. After grinding the artificial molar to alignment with the natural teeth and changing the plate, and in no other way, the patient left satisfied.

The next case is that of a physician, showing natural tolerance, for whom I made a bridge of a lateral and a cuspid supported by a crown on a loose first bicuspid, the near central being too loose to support one end.

The bridge was made with a gold plate three-eighths by three-fourths of an inch extending from the palatal surface of the lateral over and engaging the ridge. Clearly this is not clean, the bridge and plate moving with the loose bicuspid supporting it; nevertheless there is no sign of an inflamed area around this plate, and after three years it is still worn comfortably.

A case showing natural adaptability is a bridge for a young man consisting of the upper four incisors supported by the cuspids. In this case the absorption and consequent gum shrinkage was extreme, requiring the use of gum teeth, which could not be made to touch the gum and bear the normal relation to the lower teeth.

When this bridge was set temporarily, the space between the gum and the gold backing was at least three-sixteenths of an inch.

After it had been worn for two days the frænum was considerably lacerated by the porcelain gum being too high. This was remedied by grinding. Later the patient was annoyed by liquids and small particles of food passing between the gum and bridge, over the latter and under the lip. Three days later the patient reported no further annoyance from the space. Upon examination I found that the gum, without sign of inflammatory process, had extended out to the bridge until the space was entirely closed.

In conclusion, I beg to state that it has long been observed that the absorption of process is less when artificial teeth are substituted than when they are not.

This fact would tend to suggest the possibility of the process, under these bicuspids, increasing in density under the stimulus rather than its being absorbed. In my opinion this is not a space for a removable bridge. The application of this denture presupposes the two bicuspids the only missing teeth.

If the masticating area of six molars and two bicuspids is taken in the aggregate and compared to the masticating surface of these two bicuspids, the strain upon the latter in comparison will be found to be small. There is present this evening a young man wearing a denture like the one described, which I will be glad to have the members examine.

Adjourned.

OTTO E. INGLIS,
*Editor Academy of Stomatology.*

